# Examining body appreciation in six countries: The impact of age and sociocultural pressure

**DOI:** 10.1371/journal.pone.0306913

**Published:** 2024-07-31

**Authors:** Louise Nicole Hanson, Alexis Gott, Megan Tomsett, Elozino Useh, Eloise Yeadon-Caiger, Rachel Clay, Jiamin Fan, Kennice Hui, Hongdi Wang, Elizabeth Helen Evans, Dorothy Cowie, Lynda Gaynor Boothroyd

**Affiliations:** Department of Psychology, Durham University, Durham, United Kingdom; Union College, UNITED STATES OF AMERICA

## Abstract

Previous research on body appreciation across the lifespan has produced conflicting results that it increases with age, decreases with age, or is generally stable with an increase in women over 50-years-old. Furthermore, most of the research has been conducted in White, Western populations. Cross-cultural research suggests that both Chinese and African women experience similar sociocultural pressures as White Western women, and that appearance ideals are shifting to resemble a more Western ideal. We cross-sectionally and cross-culturally examined body appreciation across the lifespan, recruiting White Western women (UK, USA, Canada, and Australia), Black Nigerian women, and Chinese women. 1186 women aged 18–80 completed measures of body appreciation, internalisation of thin and athletic ideals, and perceived sociocultural pressure. Body appreciation did not vary with age in women from any country. Nigerian women reported the highest body appreciation, and Western women the lowest. Higher thin/athletic ideal internalisation, and higher perceived sociocultural pressure were significantly associated with lower body appreciation in all countries and age-groups. Overall, our findings indicate that although levels of body appreciation differ drastically between ethnicities and cultures, it is generally stable across age, and shows cross-culturally robust relationships between sociocultural internalisation and pressure.

## 1. Introduction

Body image is a multifaceted and complex phenomenon encapsulating how we think, behave, and feel about our body [1, 2], including how it is influenced by gender, culture, and sociocultural influences. To date, most body image research has focused on young, White, Western women. A much smaller volume of research has investigated body image in a cross-cultural context, and in women of a wider age range. Further, most work has focussed on negative, rather than positive body image. Positive body image is argued to be more than the absence of body dissatisfaction–it is a broader concept which includes body appreciation, high body esteem, functional body image, and body satisfaction [3–5]. Higher levels of positive body image are associated with improved quality of life [6, 7], and decreased internalisation of appearance ideals [8]. The current study focuses on body appreciation, a concept defined by Tylka and Wood-Barcalow which encapsulates positive thoughts and feelings regarding one’s own body and promotes healthy body-related behaviours [9].

In a sociocultural context, the Tripartite Influence Model (TIM) [2] posits that body image is influenced by three main sources: family, peers, and the media. However, the original model is intended to account for body dissatisfaction, and does not feature body appreciation [2, 10]. Recently, some studies have investigated body appreciation as a moderator between sociocultural pressures and body dissatisfaction and have found that it moderates this relationship [11]. It furthermore acts as a protective factor against body dissatisfaction [10] and reduces likelihood of future risky health behaviours [9, 10, 12, 13]. Additionally, body appreciation is argued to be distinct from body dissatisfaction and thus may function differently in the context of sociocultural variables [9]. Furthermore, in their review of body appreciation literature, Linardon et al., [14] recommended that future research focus on body appreciation and the factors underpinning it in non-Western populations, and across the lifespan. The current study therefore intends to explore this relationship by determining how body appreciation is predicted by the facets of the TIM across women of different cultures and ages.

In a review of global body image literature, Rodgers et al. [15] highlight the disparity of literature in non-Western populations compared to English-speaking Western populations, especially in the positive body image field. Research in Africa is the least populous, while research in English-speaking Western countries makes up the majority [16, 17]. They recommend including more diverse groups, especially in Africa and the Middle East, and increasing the range of ages included in body image research. Therefore, in this study, we consider how adult women’s body appreciation is related to sociocultural pressures and internalisation of physical ideals across age in three different cultural contexts: White Anglophone countries, China, and Nigeria.

### 1.1 Sociocultural influences on body image

There is strong evidence for the impact of sociocultural pressure on body image across the lifespan and across cultures, although the majority of research in this area has been conducted on young, White, Western samples. These studies have improved our understanding of how perceived pressure from family, peers, and the media influence our body ideals, and subsequently our body dissatisfaction, but less is known about how the TIM relates to body appreciation. In an ethnically diverse sample, Burke et al., [18] found that while mean levels of perceived pressure differed based on ethnicity, the strength of the TIM’s pathways were the same, indicating that culturally diverse women in the USA experienced similar sociocultural pressures, and that this reliably predicted body dissatisfaction levels.

The relationship between sociocultural pressure and body dissatisfaction is often mediated by thin-ideal internalisation, which has been reported across the lifespan [19–22] and is a main predictor of body dissatisfaction [23, 24] and disordered eating [25, 26]. Conversely, body appreciation has been shown to act as a protective factor against thin-ideal internalisation and disordered eating in adolescent girls, and against media pressures and disordered eating in adolescent boys [11], but in adults this relationship may be flipped as increased perceived media pressure has been suggested to reduce body appreciation [27]. Furthermore, with increasing globalisation of Western media which has been suggested to be a main factor in the increase in preference for thinner bodies in non-Western populations [28], it is important that we consider how sociocultural factors are related to body appreciation across cultures.

### 1.2 Body image and age

Research conducted with older participants has, thus far, found conflicting evidence for how women’s body image develops through the lifespan. Tiggemann and McCourt [5] recruited women aged 18 to 75, and found that, while body satisfaction/dissatisfaction were generally stable across age, women over the age of 50 had significantly higher body appreciation. The authors define this difference as body shape and weight (dis)satisfaction being stable across the lifespan, while appreciation of body functionality and health increases. As such, women who were older appreciated their bodies significantly more than younger women but remained as satisfied/dissatisfied. The authors theorise that this is due to older women’s bodies increasingly diverging from the societal ideal and therefore being ‘forced’ to accept and appreciate their bodies for their functionality and health over their physical appearance. Gagne et al. [29] found that over 70% of women over 50 reported being more dissatisfied with their current body compared to when they were younger, and 79% felt that their body shape played an important role in their self-perception. However, the women in this study were asked to think retrospectively about their younger bodies, while Tylka and McCourt compared women across the lifespan on how they felt in the present. As such, it may be the case that women think more fondly about their more youthful bodies than they did when they were inhabiting them. Additionally, previous research has suggested that body (dis)satisfaction remains stable across all ages while body appreciation increases [5]. As such, while a majority of their sample expressed high body dissatisfaction, they may have also expressed high body appreciation if it had been measured. This seems unlikely as 62% felt that their body shape or weight negatively affected their lives, which is not in keeping with high body appreciation. This finding is in line with More et al., [4] who suggest that body dissatisfaction and body appreciation represent a body image continuum, as opposed to separate constructs. However, More and colleagues only measured young participants (18–30) and so this may suggest that body appreciation and body dissatisfaction are a continuum in early life but diverge as separate constructs in later life. Two further studies have found that older women were significantly more satisfied with their body size and shape than younger women [30, 31], highlighting that body image in older women is not well understood.

Frederick and colleagues [32] propose that because older individuals are further from the youthful body ideal, due to different social roles (parenthood, employment) and higher likelihood of being in a long-term committed relationship, older individuals experience less sociocultural pressure. Other findings by Tiggemann and Lynch [33] support this theory, reporting that while body dissatisfaction remains stable, self-objectification, body monitoring, appearance anxiety and disordered eating symptomology were all significantly higher in women aged 20–29 than women aged 30–69, and lowest in women over 70 [33]. McKinley [34] found in a 10-year longitudinal study that body esteem increased across time, while body surveillance and body shame decreased. They further suggest that changes in body image are more likely to occur at times of important life transitions (e.g., leaving University for employment, onset of menopause) rather than a gradual change over time. Their results provide some support for this hypothesis as the younger women (18–20 years old at time 1 and 27–30 at time 2) reported larger changes in body esteem than the older women (40–58 at time 1 and 50–68 at time 2), and indeed the older women showed no change in body surveillance or shame while the younger women reported significant reductions. These findings suggest that body image across the lifespan in adult women may not be a linear relationship, but that it may change quickly at times of important life changes and remain more stable at times of relative calm.

### 1.3 Body image cross-culturally

Most body image research has been conducted on individuals in culturally Western countries such as the United States of America (USA), the United Kingdom (UK), Australia, and Canada. Recently, some research has been conducted in countries which have markedly different appearance cultures, such as China (where thin ideals may be stronger than in the West) and West Africa (where thin ideals may be weaker than in the West) [35]. Although there are fewer studies in these countries than in Western samples, and the sample size is often smaller, existing research points to the global diversity of appearance pressures experienced by women.

Studies in China have indicated that Chinese women report strong internalisation of the thin ideal [36–42] and cross-cultural comparisons suggest that Chinese women are more dissatisfied with their bodies than women from Croatia [41], but more satisfied than women from Japan and the USA [43]. Other research has shown that Chinese women believe being closer to the (Chinese) cultural ideal of beauty has socioeconomic benefits such as higher likelihood of employment, being able to use appearance to gain material benefits, and attracting male romantic partners [44]. Despite a growing literature investigating body image within China, few studies to date have directly compared Chinese women to Western women, and to our knowledge none have compared Chinese and African women. Some studies have considered Eastern Asian women living in Western countries, and have found for instance that Asian American women report less body dissatisfaction than White American women [18, 45, 46], but may be more focussed on physical features unrelated to body shape or size [18, 47]. While most research on Chinese women has focussed on body dissatisfaction, some recent studies have examined the validity of the Body Appreciation Scale– 2 [9] in this population, and have found comparable levels of body appreciation to women from other countries when comparing between studies [48, 49]. Finally, no studies have compared Chinese women’s body image across the lifespan, and the few studies which have looked at older Chinese adults report that they experience body dissatisfaction through the TIM’s pathway, similarly to younger Chinese adults [50, 51], highlighting the need for more research on body appreciation across the lifespan in Chinese adults.

Research in several African countries suggests that body ideals in Africa are significantly different than those in Europe and Asia, with the ideal body size being larger [35, 52]. However, research in African countries (and specifically in Nigeria for our purposes) is sparse compared to that focusing on Western and Eastern Asian populations [53]. Macia et al., [54] completed a study in Senegal which considered individual’s body image across the lifespan and reported that older Senegalese adults were less satisfied with their body than young adults. However, this study used only four questions adapted from a different study on Swedish adults [55], and reported a high degree of appearance satisfaction, even among the ‘less satisfied’ older age groups. Another West African study from Ghana found that higher belief in Afrocentric values mediated the relationship between body image satisfaction and psychological wellbeing [56]. The authors suggest that internalisation of African values and culture acts as a protective factor against body dissatisfaction as these promote a larger, healthier body. However, studies which have looked at urban Nigerian youth have found that between 36.7% and 55% of young Nigerians report body dissatisfaction [57, 58], and that rural Nigerian adolescents have a significantly better body image [57]. The author suggests that this is due to higher internalisation of traditional Afrocentric values in rural populations, and more exposure to Western media in urban populations. Further research has suggested that urban Nigerian women’s body ideal is shifting to resemble a more Western ideal (thin body, light skin, long and straight hair) [59, 60], compared to urban women from other African nations such as Kenya [61]. Additionally, all studies which have considered body image in Nigerian women have used measures of negative body image and body dissatisfaction. It should be noted that strong identification with Black African culture has been shown to reduce body dissatisfaction and promote body appreciation among African American women [62]. Despite this, only one study has looked into women across the lifespan in Western African countries. It is therefore especially important to further our knowledge of body appreciation and the role of sociocultural factors in Black African women of all ages.

### 1.4 Current study

The current study aimed to understand how body appreciation differed across age in three different cultures, and how sociocultural pressure influenced body appreciation across these populations. We therefore recruited participants from Western countries (Australia, Canada, United Kingdom, United States of America), China, and Nigeria to fill in a series of questionnaires on body appreciation and sociocultural pressure. We first hypothesised that older participants would report greater body appreciation across all groups. Drawing on previous research, we further expected that Black Nigerian women would report the highest levels of body appreciation, and White Western women would report the lowest levels. Second, we expected that older participants would report lower thin/athletic ideal internalisation and perceived sociocultural appearance pressure, and that this would be the same across ethnicities. Our final hypothesis was that high thin ideal internalisation and perceived sociocultural pressure would be associated with lower body appreciation in all cultures.

## 2. Methods

### 2.1 Ethics

Ethical approval was gained from Durham University’s Psychology Department Ethics Committee. After reading an online information sheet explaining the study’s methods and purpose, participants completed an online consent form on which they confirmed they had read and understood the information sheet and privacy notice, and agreed to participate. If participants indicated they did not consent they did not proceed further. Participants also viewed a debrief statement once they had completed all questions, in which the study’s purpose was reiterated and a link was provided to a body image support website.

### 2.2 Participants

Data were collected in two waves. Wave 1 took place from 20^th^ October 2021 to 1^st^ March 2022 and included Black Nigerian women and some of the White Western women. Wave 2 took place from 10^th^ October 2022 to 1^st^ March 2023 and included the Eastern Asian Chinese women and some of the White Western women.

Participants were recruited to this fully online study via: Durham University’s departmental participant pool (Durham university students); word of mouth within the UK, China and Nigeria; posters which were posted around Durham (England) and Glasgow (Scotland); and online promotion on popular social media platforms (Facebook, Twitter, Weibo) for all countries. The study was mainly advertised in urban areas, with focus points of Durham, Glasgow, and Washington DC for the Western population, Lagos for the Nigerian population, and Hohhot and Shanghai for the Chinese population. A total of 1393 women were recruited with an age range of 18–80 and a mean age of 34.89 (SD 14.77).

Participants completed the questionnaires as part of a larger cross-sectional study exploring body appreciation, relationship status and satisfaction, and pregnancy. The current study reports relationships amongst body appreciation, sociocultural factors, age, and cross-cultural variables. All participants were recruited using similar methods (social media, word of mouth through an author), with the exception of posters in the UK (these accounted for a small number of the older UK sample) which were not distributed in other countries.

Participants were included in the current analyses if they reported their current country of residence as Australia, Canada, United Kingdom, United States of America, China, or Nigeria. We then split participants into three groups based on country of residence and ethnicity. The White Western group consisted of women reporting White ethnicity and currently living in Australia, Canada, the UK or the US. The Nigerian group was based on women reporting Black/African ethnicity and currently living in Nigeria. The Chinese group was women currently living in China reporting Eastern Asian ethnicity. All other combinations were removed. This left a final sample of 1186 women; 811 White Western participants aged 18–76, mean 36.04 (SD 15.96), 246 Black Nigerian participants aged 18–57, mean 32.17 (SD 10.85), and 129 Eastern Asian Chinese participants aged 18–80, mean 37.36 (SD 11.52).

### 2.3 Measures

Participants completed the following questionnaires in order unless specified:

#### 2.3.1 Demographic questions

Participants were asked to indicate their age in years, gender, sexual orientation, ethnicity, country of residence, and relationship status, at the start of the study.

#### 2.3.2 Body appreciation

Participants completed the Body Appreciation Scale– 2 (BAS-2) [49, 63] as a measure of positive body image. The BAS-2 contains 10 items concerning acceptance of (e.g. “I appreciate the different and unique characteristics of my body”), positive thoughts about (e.g. “I feel good about my body”), and respect for (e.g. “I respect my body”) one’s body. All items are forward scored on a 5-point Likert-style scale from ‘Never’ to ‘Always’ where a high average score indicated higher body appreciation. The questionnaire has been shown to have good reliability and validity [63]. It has also been validated in China [48, 49], in other non-Western countries such as Japan, Poland, Serbia, and Iran [64] and in Zimbabwe [65] indicating that it is suitable for use in this study. In the current study, the internal consistency values (Cronbach’s Alpha) were .94 for White Western participants, .94 for Black Nigerian participants, and .92 for Chinese participants.

#### 2.3.3 Sociocultural pressures and thin-ideal internalisation

Participants completed the Sociocultural Attitudes Towards Appearance Questionnaire– 4 (SATAQ-4) [41, 66], a 22 item scale split into five subscales concerning: internalisation of thin ideals (e.g. “I want my body to look very thin”), internalisation of the athletic/muscular ideal (e.g. “I think a lot about looking muscular”), pressure from family (e.g. “Family members encourage me to get in better shape”), pressure from peers (e.g. “My peers encourage me to get thinner”), and pressure from the media (e.g. “I feel pressure from the media to improve my appearance”) to obtain and maintain an idealised body. Items are forward scored on a 5-point Likert-style scale from ‘definitely disagree’ to ‘definitely agree’ where a high average score indicates higher internalisation of the thin/muscular ideal, and higher perceived pressure from family, peers, and the media. This questionnaire has been shown to have good reliability and validity in the UK [66], Brazil [67], and China [68], indicating that it has good cross-cultural validity. Previous versions of the SATAQ have been shown to have good reliability in Ghana [69] and in women of African origin [70]. Internal consistency values for each subscale by country are listed in *Table 1*.

**Table 1:** Internal consistency (Cronbach’s Alpha) for each subscale of the SATAQ-4 by study group.

	Thin ideal internalisation	Athletic ideal internalisation	Pressure from family	Pressure from peers	Pressure from media
**Black Nigerian**	.67	.85	.85	.88	.90
**Eastern Asian Chinese**	.80	.79	.86	.90	.96
**White Western**	.81	.90	.90	.91	.95

### 2.4 Procedure

Participants were directed to follow a link or scan a QR code which took them directly to the study hosted by Qualtrics^TM^, an online research platform. Separate links were provided for English vs Mandarin Chinese versions of the questionnaire; Black Nigerian and White Western women completed the English version, and Eastern Asian/Chinese women, the Chinese version.

After reading the information sheet and privacy notice and clicking a button to ‘sign’ the consent form, participants provided demographic information and completed the questionnaires described under Measures.

### 2.5 Statistical analysis

In advance of the main analyses, we conducted tests of missingness to determine the potential influence of missing data. We found that Black Nigerian women were more likely to have missing data on the BAS-2, but otherwise the data was missing at random. As such, we used mean imputation for missing items where participants had completed over half of the questions in a given scale. We then use pairwise deletion for analyses where participants have missed an entire scale.

To answer our main research questions, we conducted a series of multiple linear regressions. To test our first hypothesis that with increased participant age there would be a significant increase in body appreciation for all women, and that this would be strongest for Black Nigerian woman, and weakest for White Western women, we conducted two linear regressions, using age, ethnicity, and their interaction as predictor variables. For our second hypothesis, that with increased participant age there would be a significant decrease in thin/athletic ideal internalisation and perceived sociocultural pressure, and this would be the same across ethnicities, we conducted five linear regressions (for each subscale of the SATAQ-4) using age, ethnicity, and their interaction as predictor variables. Finally, for our third hypothesis that high thin ideal internalisation and perceived sociocultural pressure would be associated with lower body appreciation, we ran five correlations (one for each subscale of the SATAQ-4).

Summary statistics and zero-order correlations for all variables with the full sample are shown in Table 2. Other than age and body appreciation, all variables were significantly correlated with each other.

**Table 2:** Tabulated correlations (Pearson’s r) for all variables in the full sample (N = 966).

	Ethnicity	Mean	Standard Deviation	1	2	3	4	5	6
1. Age (years)	Black Nigerian	32.17	10.85						
	Chinese	37.36	11.52						
	White Western	36.04	15.96						
2. BAS-2	Black Nigerian	4.35	0.71	.07					
	Chinese	3.90	0.68	.28**					
	White Western	3.18	0.83	.07*					
3. SATAQ-4 (athletic)	Black Nigerian	2.21	0.76	-.10	-.07				
	Chinese	3.49	0.69	.04	.11				
	White Western	2.63	0.87	-.42**	-.18**				
4. SATAQ-4 (thin)	Black Nigerian	2.04	0.90	-.06	-.18**	.78**			
	Chinese	2.90	0.74	.05	-.01	.72**			
	White Western	2.50	1.05	-.39**	-.21**	.86**			
5. SATAQ-4 (family)	Black Nigerian	2.19	1.11	-.24**	-.27**	.31**	.35**		
	Chinese	2.46	0.96	.02	-.35**	.30**	.40**		
	White Western	2.30	1.20	-.22**	-.27**	.19**	.21**		
6. SATAQ-4 (peers)	Black Nigerian	2.11	1.11	-.20**	-.14*	.29**	.31**	.64**	
	Chinese	2.76	1.06	.05	-.31**	.27**	.38**	.61**	
	White Western	1.88	1.00	-.12**	-.27**	.26**	.28**	.47**	
7. SATAQ-4 (media)	Black Nigerian	2.41	1.26	-.16*	-.46**	.24**	.35**	.48**	.52**
	Chinese	2.66	1.20	-.19*	-.25**	.21-	.19*	.50**	.67**
	White Western	3.64	1.27	-.24**	-.35**	.39**	.34**	.34**	.34**

BMI = Body Mass Index; BAS-2 = Body Appreciation Scale 2; SATAQ-4 = Sociocultural Attitudes Towards Appearance Questionnaire 4. *p < .05, **p < .01

## 3. Results

Linear Regression models were used to test for possible effects of age, ethnicity, sociocultural attitudes and thin/athletic internalisation on body appreciation. Analyses were conducted using R version 4.3.0 [71] and data and code can be found in the Supporting Information.

Descriptive statistics broken down by study group are shown in *Table 3*.

**Table 3:** Descriptive statistics including mean age, BAS-2, and SATAQ-4 for each study group.

	Number of ppts	Mean Age (years)	BAS-2	SATAQ-4 (athletic)	SATAQ-4 (thin)	SATAQ-4 (family)	SATAQ-4 (peers)	SATAQ-4 (media)
Black Nigerian	246	32.17	4.35	2.21	2.04	2.19	2.11	2.41
Eastern Asian Chinese	129	37.36	3.90	3.49	2.90	2.46	2.76	2.66
White Western	811	36.04	3.18	2.63	2.50	2.30	1.88	3.64

### 3.1 Body appreciation would be higher in older women, with Black Nigerian women reporting the highest body appreciation, and White Western women reporting the lowest

**Fig 1:**
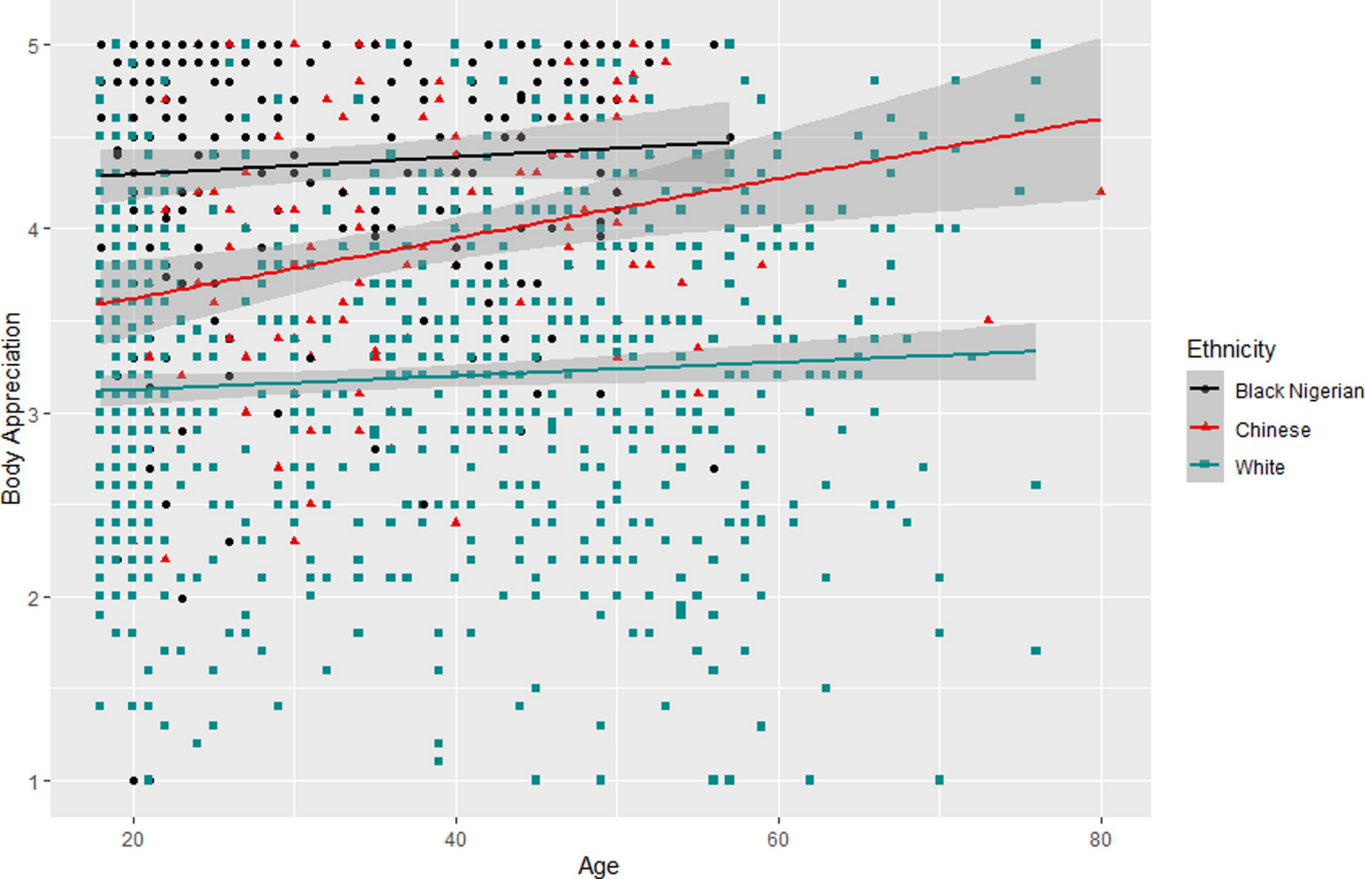
Scatterplot showing the relationships of age and ethnicity with body appreciation. Black circles represent Black Nigerian women; Red triangles represent Eastern Asian Chinese women; and cyan squares represent White Western women. Lines indicate linear model.

Data were entered into linear regression models with BAS-2 as the outcome variable, and age (Table 4, Model 1), and ethnicity and the age x ethnicity interaction (Model 2) as predictors. In model 1 we found no significant effect of age on BAS-2 score (p = .342). In Model 2, we conducted a linear regression with Black Nigerian women as the reference category and found that there was no significant effect of age, but both Chinese and White Western women had lower body appreciation than Black Nigerian women, with White Western women scoring the lowest (Model 2: F(5, 1173) = 90.66, p < .001, R^2^_Adjusted_ = .28; see *Table 4*). There were no significant interaction effects, although Fig 1 suggests that Chinese women may show a more positive trend between age and body appreciation than other groups in a larger sample (note the width of the CI shading in the upper age range).

**Table 4:** Linear regression models for effects of age, ethnicity, and their interaction on BAS-2. Ethnicity entered as a categorical predictor with Black as reference category.

	Model 1				Model 2			
	β	SE	t	p	β	SE	t	p
(Intercept)	3.44	0.07	48.64	< .001	4.20	0.16	26.37	< .001
Age	0.03	0.002	0.95	.342	0.07	0.005	1.01	.315
**Ethnicity (Chinese)**					**-0.30**	**0.29**	**-3.16**	**.002**
**Ethnicity (White)**					**-0.57**	**0.17**	**-6.63**	**< .001**
Age: Ethnicity (Chinese)					0.15	0.01	1.51	.132
Age: Ethnicity (White)					-0.02	0.005	-0.20	.841

### 3.2 Thin ideal internalisation and perceived sociocultural pressure would be lower in older women, with no effect of ethnicity

**Fig 2:**
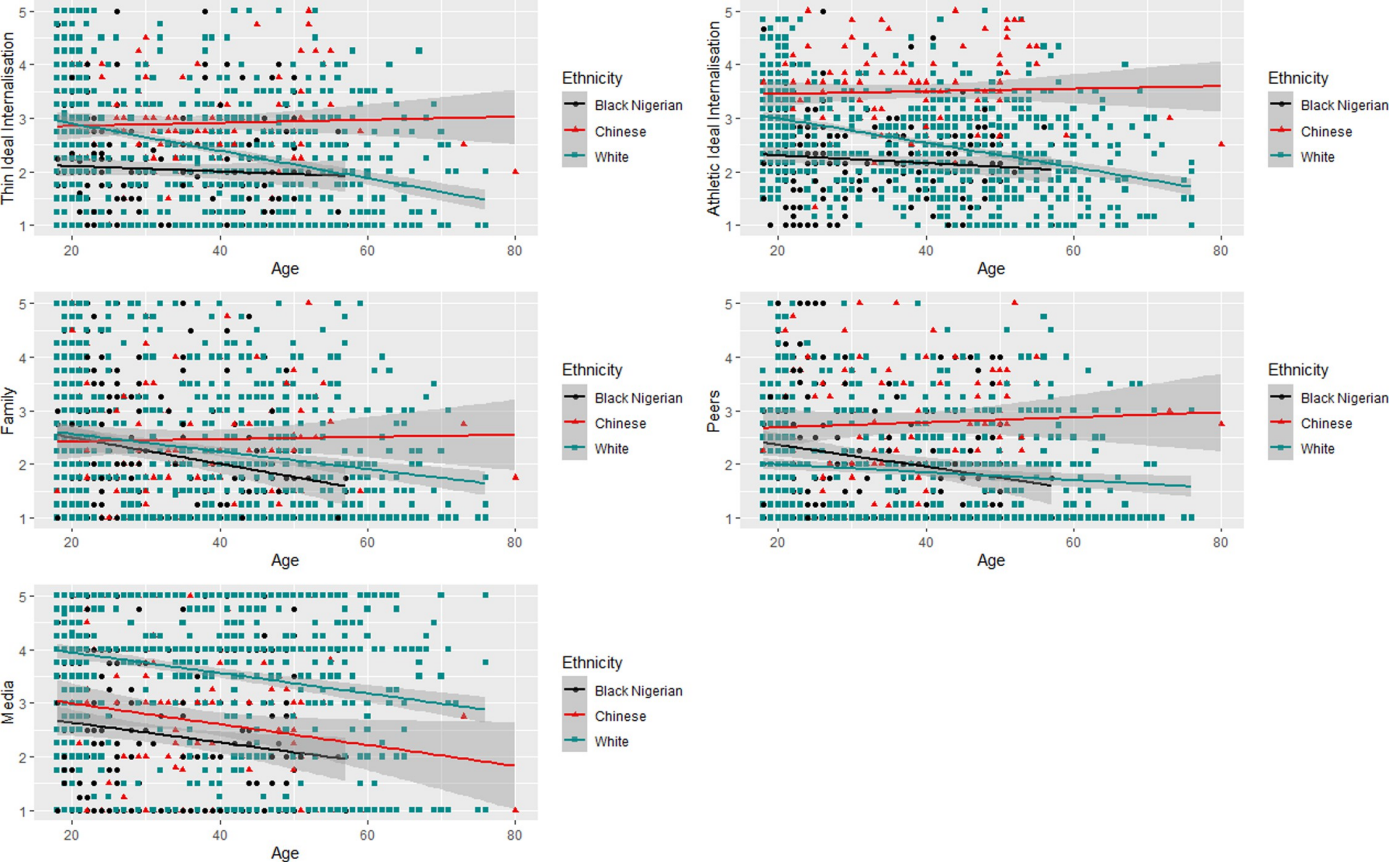
Scatterplots indicating the effect of age and ethnicity on SATAQ-4 subscales. Top left: Athletic ideal internalisation; Top right: Thin ideal internalisation; Middle left: Pressure from family; Middle right: Pressure from peers; Bottom left: Pressure from the Media. Black circles represent Black Nigerian women; Red triangles represent Eastern Asian Chinese women; and cyan squares represent White Western women. Lines indicate linear model.

Data were entered into linear regression models with SATAQ-4 subscales (internalisation of the thin ideal, internalisation of the athletic ideal, perceived pressure from family, perceived pressure from peers, perceived pressure from the media) as the outcome variables, and age, and ethnicity as predictors. We found that perceived sociocultural pressure from family, peers, and the media decreased significantly as age increased (see *Fig 2*), but there was no significant effect of age on thin-ideal or athletic-ideal internalisation (See *Table 5*). With Black Nigerian women as the reference category, we found that there was no significant main effect of ethnicity on perceived pressure from family, but that it remained stable across age for Chinese women while it decreased with age for White Western and Black Nigerian women (F(5, 1101) = 11.56, p < .001, R^2^_Adjusted_ = .05). White Western women experienced significantly higher pressure from the media (F(5, 1095) = 131.5, p < .001, R^2^_Adjusted_ = .37) and significantly lower pressure from peers (F(5, 1097) = 107.9, p < .001, R^2^_Adjusted_ = .33), and this interacted with age–Black Nigerian women reported less perceived pressure from peers as age increased, while this remained generally stable for White Western and Chinese women. Finally, we found that Chinese women reported the highest thin and athletic ideal internalisation followed by Western women, both of whom were significantly higher than Nigerian women, and that there was a significant interaction between age and White ethnicity. As shown in Fig 2, White Western women’s thin (F(5, 1101) = 43.82, p < .001, R^2^_Adjusted_ = .16) and athletic (F(5, 1103) = 76.94, p < .001, R^2^_Adjusted_ = .26) ideal internalisation was lower with higher age, while no such relationship was observed for the other groups (See *Fig 2*).

**Table 5:** Linear regression models for effects of age, ethnicity, and their interaction on SATAQ-4 subscales.

	β	SE	t	p
**SATAQ-4 Thin Ideal Internalisation**				
(Intercept)	2.20	0.19	11.30	< .001
Age	-0.07	0.006	-0.87	.383
Ethnicity (Chinese)	0.18	0.35	1.71	.087
**Ethnicity (White)**	**0.55**	**0.21**	**5.73**	**< .001**
Age: Ethnicity (Chinese)	0.09	0.01	0.85	.396
**Age: Ethnicity (White)**	**-0.43**	**0.01**	**-3.39**	**< .001**
**SATAQ-4 Athletic Ideal Internalisation**				
(Intercept)	2.44	0.16	15.08	< .001
Age	-0.12	0.005	-1.51	.132
**Ethnicity (Chinese)**	**0.33**	**0.29**	**3.32**	**< .001**
**Ethnicity (White)**	**0.52**	**0.18**	**5.73**	**< .001**
Age: Ethnicity (Chinese)	0.13	0.008	1.25	.213
**Age: Ethnicity (White)**	**-0.37**	**0.005**	**-3.10**	**.002**
**SATAQ-4 Family**				
(Intercept)	2.99	0.24	12.58	< .001
**Age**	**-0.31**	**0.01**	**-3.54**	**< .001**
Ethnicity (Chinese)	-0.16	0.42	-1.44	.150
Ethnicity (White)	-0.04	0.26	-0.36	.716
**Age: Ethnicity (Chinese)**	**0.28**	**0.01**	**2.36**	**.018**
Age: Ethnicity (White)	0.15	0.01	1.10	.273
**SATAQ-4 Peers**				
(Intercept)	2.77	0.21	13.04	< .001
**Age**	**-0.28**	**0.01**	**-3.29**	**.001**
Ethnicity (Chinese)	-0.05	0.38	-0.47	.636
**Ethnicity (White)**	**-0.27**	**0.23**	**-2.72**	**.007**
**Age: Ethnicity (Chinese)**	**0.29**	**0.01**	**2.49**	**.013**
**Age: Ethnicity (White)**	**0.26**	**0.01**	**1.98**	**.048**
**SATAQ-4 Media**				
(Intercept)	3.00	0.26	11.72	< .001
**Age**	**-0.20**	**0.01**	**-2.46**	**.014**
Ethnicity (Chinese)	0.09	0.46	0.83	.406
**Ethnicity (White)**	**0.45**	**0.28**	**4.74**	**< .001**
Age: Ethnicity (Chinese)	-0.01	0.01	-0.08	.937
Age: Ethnicity (White)	-0.01	0.01	-0.08	.939

### 3.3 High thin ideal internalisation and perceived sociocultural pressure would be associated with lower body appreciation

We used Pearson’s correlations to test our third hypothesis, looking at the relationship between BAS-2 and each SATAQ-4 subscales (internalisation of the thin ideal, internalisation of the athletic ideal, perceived pressure from family, perceived pressure from peers, perceived pressure from the media). For all subscales there was a negative relationship between ideal body internalisation/sociocultural pressure and body appreciation, indicating that higher internalisation of thin ideal (r(1098) = -0.22, p < .001), higher internalisation of the athletic ideal (r(1100) = -0.15, p = .001) and higher perceived sociocultural pressure from family (r(1098) = -0.24, p < .001), peers (r(1101) = -0.12, p < .001), and the media (r(1099) = -0.49, p < .001) were associated with lower body appreciation (see *Table 2*). There were no interactions with ethnicity (see Supporting Information).

## 4. Discussion

The aim of the present study was to determine how body appreciation was associated with age in women from three different cultures, and how body appreciation is associated with internalisation of thin and athletic ideals and perceived sociocultural pressure.

Our first hypothesis was that body appreciation would be higher in older women, that Black Nigerian women would report the highest body appreciation, and White Western women would report the lowest was partially supported. We found no significant difference in body satisfaction between women of different ages. These results are in contrast with previous research which found that women’s body appreciation increased across the lifespan [5, 30, 31, 34]. We found that Black Nigerian women had the highest body appreciation, followed by Eastern Asian Chinese women, and White Western women had the lowest body appreciation. This is in keeping with previous research which has found that Chinese women are more satisfied with their bodies than Western populations [43]. In additional analyses, we found that ethnicity remained the strongest predictor of body appreciation even when considering its interaction with perceived sociocultural pressure and age, and when controlling for BMI. This suggests that ethnicity and culture are important influences for body appreciation and can act as protective factors which promote positive body image.

Our second hypothesis that older women would report significantly lower thin ideal internalisation and perceived sociocultural pressure than younger women, and that this would be the same across study groups, was partially supported. We found that older White Western and Black Nigerian women reported significantly lower thin-ideal internalisation than younger women, while there was no relationship between thin-ideal internalisation and age-group in Chinese women. For women from all cultures, older women reported lower perceived sociocultural pressure from all sources, although White Western women experienced less perceived pressure from peers than Chinese women and more from media than Black Nigerian and Chinese women. Our results also showed an association between perceived sociocultural pressure and ethnicity, suggesting that Black Nigerian women experienced the lowest sociocultural pressure, and that Chinese women reported the most pressure. This finding is in keeping with studies from China which have found that Chinese women have strong internalisation of the thin ideal [36, 38–42].

Our third hypothesis that high thin ideal internalisation and perceived sociocultural pressure would predict with lower body appreciation was supported by our results. We found that high internalisation of the thin ideal, and high perceived pressure from family, peers, and the media, all were associated with significantly lower body appreciation. This provides evidence for the TIM [2] using body appreciation instead of body dissatisfaction. Given that body appreciation may be a separate concept from body dissatisfaction [3–5] and previous research has mainly considered how sociocultural pressures and thin ideal internalisation influence body dissatisfaction [10], our study has shown the inverse relationship exists with body appreciation, expanding our understanding of how body appreciation interacts with the TIM to predict the way we feel about our body. We found that across age and culture, perceived sociocultural pressure to obtain a culturally ideal body was a strong predictor of body appreciation, specifically that those who reported higher perceived sociocultural pressure had significantly lower body appreciation than those who reported low sociocultural pressure.

### 4.1 Limitations and future research

It is important to note that while we had a large population of women from across the lifespan, our age range remained skewed at the bottom end, with a large proportion of our sample aged between 18–30 years, and smaller numbers of older participants. In a fully representative sample we would predict a drop in participation after 50 years as the majority of the UK and Chinese populations are aged 20–50 years [72, 73], but our sample declines prior to that, at around 30 years. Therefore, further research should seek to recruit more women in the older age groups to get a fully representative picture of women’s body appreciation across the lifespan. This could be achieved through use of appropriate reimbursement for time, or recruitment tactics which may be more accessible to older individuals, with word-of-mouth [74], mass mailing and email [75], and recruitment through community settings [76] suggested as the most acceptable tactics for recruitment of an older population.

Furthermore, we recruited a larger sample of White Western women than we did of Black Nigerian and Chinese women, and used measurement tools developed in predominantly White Western populations. Post-hoc assessment of our effect sizes suggested we had sufficient power even if all group sizes were constrained to the smallest sample (see Supporting Information ‘Power analysis’). However, the current study may not have fully captured the extent of body appreciation or sociocultural preferences in the Nigerian and Chinese populations due to cultural differences in body ideals or how they are expressed [17]. Development of measurement tools which are suitable for these populations is therefore a necessity for future research in cross-cultural contexts.

Additionally, the questionnaires that we used have been mainly tested and validated with younger populations. As such, older generations may not experience the same *kinds* of body concerns as younger women, especially given that older women are further removed from the thin ideal which can cause feelings of shame and depression [77], are more likely to participate in ‘old talk’ over ‘fat talk’ [78], can experience discrepancy between how they feel and how they look [77, 79], have changing social roles which can lead to feeling forgotten and invisible [77], and are more likely to be in a long term relationship [32]. This may mean that older women experience other kinds of concerns which are not sufficiently captured by our measures. Future research should consider the validity of using these measures with an older population and consider developing measures of body image to be used in this population.

Finally, although we advertised mainly in cities, we did not ask participants whether they were from a rural or urban area. Research has shown that body appreciation is higher and body dissatisfaction is lower in individuals living in rural areas compared to those living in urban areas [80, 81] and this effect is seen cross-culturally in Malaysia [82], South Africa [83], and Brazil [84]. As such future research should consider participants’ locality (urban/rural) when measuring body appreciation.

### 4.2 Practical implications

The results of the current study can be used when developing positive body image interventions, especially when considering a cross-cultural and lifespan perspective. For instance, body appreciation did not noticeably increase with age and as such older women may also benefit from the body image interventions which are largely targeted at younger populations. However, some aspects of sociocultural pressure and internalisation did alleviate with age; as such body image interventions aimed at older women might need to employ different foci than e.g. dissonance techniques aimed at reducing thin-ideal internalisation. However, these must be further adapted given the age by ethnicity interactions observed in some aspects of sociocultural pressures/internalisation. Specifically, it seems that Chinese women may benefit from interventions which target ideal body internalisation and perceived pressure from peers as these remained stable across age in this group, while White western women of all ages may benefit most from an intervention aimed at media pressure. Black Nigerian women tended to report the lowest sociocultural pressure, but this is not to say that they do not experience other pressure which was not captured in the scope of this study, e.g. a curvy ideal body. As such culturally specific further research is likely to be required when developing interventions for a given group.

### 4.3 Conclusion

Our study examined body appreciation and some of its predictors across the lifespan in three different cultures, finding evidence that the Tripartite Influence Model can be applied to body appreciation as well as body dissatisfaction, and in age-diverse samples. We also find that there are both similarities and differences between cultures in how body appreciation, sociocultural pressure, and thin ideal internalisation, vary across age in different cultures.

## Supporting information

S1 Dataset Raw data. (RMD)

S1 Data Main analysis using R. (RMD)

S2 Data Power analysis using R. (RMD)
